# Intestinal Vicarious Contrast Medium Excretion on Delayed Computed Tomography in Dogs with Protein‐Losing Enteropathy

**DOI:** 10.1111/vru.70039

**Published:** 2025-05-07

**Authors:** Yujin Lee, Hojung Choi, Young‐Won Lee, Kija Lee, Sooyoung Choi

**Affiliations:** ^1^ College of Veterinary Medicine and Institute of Veterinary Science Kangwon National University Chuncheon South Korea; ^2^ College of Veterinary Medicine Chungnam National University Daejeon South Korea; ^3^ College of Veterinary Medicine Kyungpook National University Daegu South Korea

**Keywords:** contrast medium, delayed computed tomography, protein‐losing enteropathy, vicarious excretion

## Abstract

The intestinal vicarious contrast medium excretion (VCME) can occur in dogs with protein‐losing enteropathy (PLE), and studies for intestinal VCME in dogs are lacking. This retrospective case‐control study aimed to assess whether intestinal VCME could be observed on delayed CT in dogs with and without PLE. Thirty dogs who underwent abdominal delayed CT in the 10 min‐delayed phase following the injection of contrast medium were enrolled. Six dogs were classified into the group with enteropathy based on imaging findings or abnormal results from cytology or histology. The six dogs had concurrent hypoalbuminemia and were diagnosed with presumed PLE. Five of the six dogs in the group with enteropathy had intestinal VCME. In the 24 dogs of the group without enteropathy, intestinal VCME was not detected on delayed CT, and VCME to the cisterna chyli was observed in one dog. The frequency of intestinal VCME was significantly higher in the group with enteropathy than in the group without enteropathy (*p* < .001). The presence of intestinal VCME on the delayed CT can be observed in dogs with PLE, and it can be used as noninvasive additional supportive evidence of canine PLE prior to histopathologic evaluation.

## 1 Introduction

Derived from the Latin word for “substitute”, vicarious contrast medium excretion (VCME) is defined as an alternative excretion of intravenously administered water‐soluble contrast medium in a way other than via renal excretion [1]. Iodinated intravenous contrast medium is rapidly concentrated within and normally excreted by the kidneys, while VCME primarily occurs in the biliary tract and can also be observed in the gastrointestinal tract [1, 2, 3]. In humans, there are several reports about this phenomenon, which resulted in opacification of the gall bladder and intestine on radiography and CT examinations [4, 5, 6]. VCME has been observed more frequently in patients with renal insufficiency in the past [7].

The most commonly encountered chronic enteropathies in dogs, characterized by persistent or recurrent gastrointestinal signs, are inflammatory bowel disease (IBD), food allergies, and small intestinal bacterial overgrowth. Protein‐losing enteropathy (PLE), which is most often a feature of IBD, is defined as a syndrome with an abnormal loss of serum protein through the gastrointestinal mucosa and is associated with mucosal injury, infectious disease, and lymphatic disorders [8, 9]. Published reports to date show that canine PLE is generally associated with lymphangiectasia, lymphoplasmacytic enteritis, lymphangitis, and crypt disease [8].

PLE is identified based on findings of panhypoproteinemia, and intestinal function tests such as serum folate and cobalamin may confirm the presence of intestinal malabsorption. The histological diagnosis is confirmed by full‐thickness or endoscopic biopsy of the intestinal tract [10]. For enteropathy, some diagnostic imaging findings, such as ultrasonographic appearances of hyperechoic striations within the mucosal layer of the small intestine, have high specificity [9, 11]. In humans, CT reveals localized intestinal lymphangiectasia (IL), which is known as the main cause of human PLE, with diffuse and nodular changes, thickening, swelling, or edema of the intestine [12]. Magnetic resonance imaging identified the primary IL as well [13]. However, the imaging findings for enteropathy are indirect evidence for PLE. To the author's knowledge, the imaging diagnosis for PLE using CT and MRI has not been reported.

PLE is understood to be the loss of serum protein into the intestinal lumen and is associated with the following pathologies: (1) increased lymphatic pressure (lymphangiectasia, lymphangitis) and (2) disease with mucosal injury (IBD, crypt disease, intestinal tumor) [14]. Increased lymphatic pressure resulting from increased venous pressure and mucosal injury due to large breaks in the epithelial permeability barrier leads to passive protein leakage into the lumen via an oncotic gradient from the interstitium to the lumen [8, 14]. It can be assumed that the contrast medium would be transported from blood serum to the intestinal lumen by a mechanism similar to that of albumin in patients with enteropathy. We hypothesized that delayed CT would reveal intestinal VCME in dogs with PLE but not in dogs without PLE and that intestinal VCME may be suggestive of PLE in dogs prior to histopathological evaluation.

This study aimed to assess whether the presence of VCME on delayed CT could be observed in dogs with or without PLE.

## 2 Materials and Methods

CT studies of client‐owned dogs from two veterinary hospitals, who presented to the Ian Animal Diagnostic Center (Seoul, Republic of Korea) between September 2016 and February 2017 and to the Kangwon Veterinary Medical Teaching Hospital (Chuncheon, Republic of Korea) between May 2017 and April 2021, were retrospectively reviewed. Dogs were included in the study if abdominal CT images were obtained at the delayed phase after intravenous administration of contrast medium. Information was collected from the clinical records on signalment, major complaints, clinical presentation, laboratory examination results, ultrasonographic findings, and indications for CT examination. Serum chemistry results, including renal values, total protein, and albumin, were recorded.

CT examinations were performed using the 16 multi‐slice scanners of Ian Animal Diagnostic Center (SOMATOM Emotion, Siemens, Munich, Germany) and Kangwon Veterinary Medical Teaching Hospital (Alexion, Toshiba, Otawara, Japan), and CT images were acquired at 120 kVp and 100–150 mA. General anesthesia was conducted by using butorphanol (0.2 mg/kg IV, Butophan, Myungmoon, Seoul, Korea) and midazolam (0.1–0.2 mg/kg IV, Midazolam, Bukwang, Seoul, Korea) for premedication and propofol (dose according to effect; 3–5 mg/kg IV, Anepol, Hana, Seoul, Korea) as induction agents. Inhalation was maintained with a mixture of isoflurane (1.5%–2%, Ifran, Hana, Seoul, Korea) and oxygen in sternal recumbency during the CT examination. For CT scans, postcontrast dual‐phase scans of the arterial and portal phases were performed using a bolus‐tracking system, starting after reaching the bolus tracking threshold at the abdominal aorta following administration of the contrast medium, iohexol (Omnipaque, GE Healthcare, Cork, Ireland, 600–700 mg I/kg) using a power injector (Salient, Imaxeon, Sydney, Australia). The delayed CT images were acquired approximately 10 min after contrast administration. For patients at risk of anesthesia, the CT scans were performed awake by wrapping the dog with a towel in sternal or lateral recumbency. Postcontrast and delayed scans were acquired following manual administration of the same contrast medium and dosage mentioned above via a cephalic vein.

All abdominal CT images were assessed in the transverse planes in a soft tissue window (width 450; level 40) by a three‐year experienced veterinarian in the veterinary radiology field. The following CT findings were recorded: the presence or absence of urinary obstruction (dilation of the renal pelvis or ureter), enteropathy (intestinal wall thickening) [15], lymphadenopathy (lymph node enlargement) [16], and VCME (gall bladder, gastrointestinal tract, etc). On multi‐phasic CT, mucosal enhancement of the intestine in the early phase is washed out in the late phase [17]. If a contrast medium is observed again in the delayed phase, intraluminal opacification can be considered. Intraluminal opacification of contrast medium within the intestine was interpreted as vicarious excretion into the small intestine.

Dogs with abnormal imaging findings on ultrasonography (intestinal wall thickening, mucosal hyperechoic striations) or CT or abnormal results from cytology or histology of the intestine were regarded to have enteropathy. Dogs without biochemical markers or imaging findings to support enteropathy were presumed not to have enteropathy. Dogs included in this study were divided into groups: those with and without enteropathy. Intestinal VCME was compared between these two groups with and without enteropathy using Fisher's exact test in SPSS (version 26.0, IBM Corp., Armonk, NY, USA).

## 3 Results

Thirty dogs were enrolled in the study, and six of the 30 dogs were included in the group with enteropathy. The six dogs had hypoalbuminemia, and one of the six dogs also exhibited azotemia. In six dogs with enteropathy, intestinal VCME was detected in five dogs, but not in the one with alimentary lymphoma. In the 24 dogs of the group without enteropathy, VCME into the cisterna chyli was detected in one dog (Table 1). The frequency of intestinal VCME was significantly higher in the group with enteropathy than in the group without enteropathy (*p* < .001). One dog with alimentary lymphoma and three of the 24 dogs without enteropathy underwent CT examinations without anesthesia owing to their risk.

**TABLE 1:** Numbers of dogs with enteropathy, hypoalbuminemia, azotemia, urinary obstruction, and vicarious contrast medium excretion (VCME).

Criteria		Enteropathy	Hypoalbuminemia	Azotemia	Urinary obstruction	VCME^a^
Dogs (*n*)	Presence	6	6	1	0	5^b^
	Absence	24	2	2	6	1^c^

^a^ The frequency of intestinal VCME was significantly higher in dogs with enteropathy than in those without enteropathy (*p* < .001).

^b^ These five dogs had intestinal VCME.

^c^ This dog had VCME to the cisterna chyli.

The group with enteropathy consisted of three castrated males and three females (two spayed). The mean age was 10.0 years (range: 4–12 years), and the mean body weight was 5.22 kg (range: 3.3–9.3 kg). Breeds were cocker spaniel, Maltese, miniature pinscher, Pekingese, poodle, and Yorkshire terrier. Clinical presentations of the dogs included diarrhea (*n* = 4), anorexia (*n* = 4), melena (*n* = 2), lethargy (*n* = 2), respiratory distress (*n* = 2), tachypnea and coughing, and abdominal distension (*n* = 1). Serum biochemistry examinations of six dogs revealed decreased concentrations of total protein (*n* = 5, range 3.4–5.0 g/dL, reference range 5.2–8.2 g/dL) and albumin (*n* = 6, range: 1.4–2.1 g/dL, reference range: 2.3–4 g/dL). Dog 2 had low serum folate levels, and the serum cobalamin and folate levels both decreased in dog 4. In additional examinations for hypoproteinemia, none of the dogs that checked serum bile acid and plasma ammonia levels as markers of hepatic dysfunction showed high concentrations, and there was no evidence of protein‐losing nephropathy in urinalysis. Examination of the pleural effusion or ascites following ultrasound‐guided abdominal paracentesis was performed in dogs 1 and 4. The fluid was diagnosed as a transudate with a low cell count and protein content. Thoracic and abdominal radiographs depicted a fissure line in the pleural space or scalloped sign and decreased abdominal serosal detail. Abdominal free fluid, hyperechoic striations or speckles in the intestinal mucosal layer (*n* = 2), and intestinal wall thickening were observed on abdominal ultrasonography.

**FIGURE 1:**
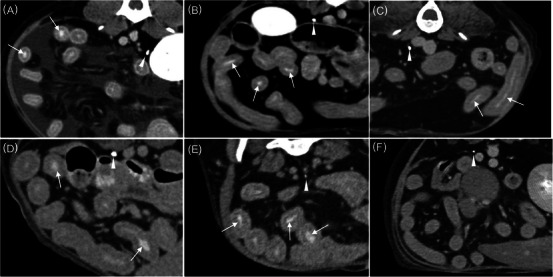
Transverse plane CT images on delay‐phase following administration of contrast medium in six dogs suspected or confirmed PLE. Dog 1 (A), dog 2 (B), dog 3 (C), dog 4 (D), dog 5 (E), and dog 6 (F). Ureteral contrast enhancement of the excretory phase can be observed (arrowheads). Note the intraluminal opacification of multiple segments of the small intestine in five dogs (A–E) (arrows) compared to that in dog 6.

CT identified ascites (*n* = 4), pleural effusion (*n* = 1), intestinal wall thickening (*n* = 4), corrugated dilation of the intestinal wall (*n* = 1), and abdominal lymphadenopathy (*n* = 4), including jejunal lymph nodes (Table 2). The amount of pleural effusion or ascites was variable. On the delayed CT, the intestinal VCME was observed in five dogs (Figure 1).

**TABLE 2:** Diagnostic imaging findings of the intestine, results of laboratory examination, and vicarious contrast medium excretion (VCME) in six dogs with enteropathy.

			Serum Biochemistry						
Number	Ultrasonography	Computed tomography	TP	Alb	BUN	Creatine	Cobalamin	Folate	Intestinal VCME
Dog 1	N/A	Corrugated dilation of the intestinal wall	Not recorded	2.1	14	0.7	N/A	N/A	Yes
Dog 2	N/A	Multifocal intestinal wall thickening, lymphadenopathy	4.1	1.6	13	0.7	N/A	Low	Yes
Dog 3	Multifocal intestinal wall thickening	Multifocal intestinal wall thickening	3.4	1.5	7	0.5	Normal	N/A	Yes
Dog 4	Multifocal intestinal wall thickening, intestinal mucosal hyperechoic striations	Multifocal intestinal wall thickening, lymphadenopathy	3.4	1.4	29	0.5	Low	Low	Yes
Dog 5	Intestinal mucosal hyperechoic striations	Lymphadenopathy	4.2	1.4	13	0.4	Normal	Normal	Yes
Dog 6	Focal intestinal wall thickening	Focal intestinal wall thickening, lymphadenopathy	5.0	2.1	78	1.1	N/A	N/A	No

*Note*: Reference range: TP, 5.2–8.2 g/dL; Alb, 2.3–4 g/dL; BUN, 7–27 mg/dL; Creatine, 0.5–1.8 mg/dL; Ca, 7.9–12 mg/dL; ^*^9.1–11.7 mg/dL; Chol: 110–320 mg/dL; ^**^135–278 mg/dL.

Abbreviations: Alb, albumin; BUN, blood urea nitrogen; Ca, calcium; Chol, cholesterol; N/A, not assessed; TP, total protein.

Histological or cytologic examination of the intestine was performed after CT examination in four dogs. The intestinal histological diagnoses for dogs 4 and 5 were confirmed as lymphoplasmacytic enteritis with multifocal lymphangiectasia. The cytologic results collected by ultrasonography‐guided fine needle aspiration of the small intestine in dog 3 revealed lymphocyte‐predominant inflammation with epithelial hyperplasia, and lymphoplasmacytic enteritis was suspected. However, an additional intestinal biopsy was not performed on dog 3. Dog 6 was diagnosed as alimentary lymphoma by cytologic evaluation.

**FIGURE 2:**
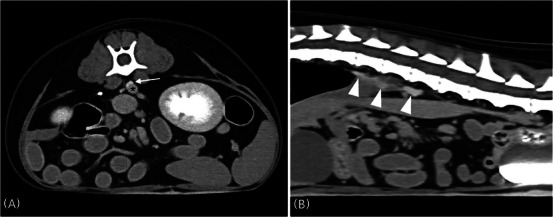
Delayed CT images of the abdomen in a dog revealed VCME into the cisterna chyli. A, Transverse delay‐phase CT image at the level of L3. The cisterna chyli (arrow) was contrast‐enhanced in a rounded and crescent‐shaped structure and lies adjacent to the aorta (asterisk). B, Sagittal delay‐phase CT image of the abdomen. The contrast‐enhanced cisterna chyli (arrowheads) and urinary bladder were visible.

Among the 24 dogs of the group without enteropathy, the most common reason for CT examinations was renal mass (Table 3). No VCME was observed in 23/24 dogs, and one dog with a portosystemic (extrahepatic splenocaval) shunt showed contrast enhancement of the cisterna chyli (Figure 2). In six of the 23 dogs without VCME, no unilateral ureteral opacification due to ureteral obstruction by renal mass (*n* = 4), urinary bladder mass (*n* = 1), or ureteral calculi (*n* = 1) was observed. Azotemia and hypoalbuminemia were identified in two of the 24 dogs, respectively, and a disorder associated with hypoalbuminemia was not definitively confirmed (Table 3).

**TABLE 3:** CT indications, hypoalbuminemia, azotemia, urinary obstruction, and vicarious contrast medium excretion (VCME) in 24 dogs without enteropathy.

A reason for CT examination	Number of dogs	Hypoalbuminemia	Azotemia	Unilateral urinary obstruction	VCME
	(*n* = 24)	(*n* = 2)	(*n* = 2)	(*n* = 6)	(*n* = 1)
Renal mass	6			4	
Mammary gland tumor	5		1		
Urinary bladder mass	2		1	1	
Hit by car	2				
Intraperitoneal mass	1				
Pancreatitis	1				
Multiple hepatic nodules	1	1			
Portosystemic shunt	1				1
Prostatic mass	1				
Spinal tumor	1				
Subcutaneous mast cell tumor	1				
Ureteral calculi	1			1	
Uterine mass	1	1			

## 4 Discussion

Before a presumed diagnosis of PLE should be made, other disorders resulting in hypoalbuminemia, such as hepatic dysfunction, protein‐losing nephropathy, whole blood loss, and systemic inflammation should be first ruled out in dogs with hypoalbuminemia. In the present study, the six dogs of the group with enteropathy had hypoalbuminemia; none showed evidence of hepatic dysfunction or protein‐losing nephropathy, and all were, therefore, could be regarded as having PLE. Delayed CT in five of six dogs revealed intraluminal opacification in the small intestine of intravenously injected water‐soluble iodinated contrast medium, indicating intestinal VCME. In contrast, intestinal VCME was not detected in any of the 24 dogs without enteropathy. The results of this study show that intestinal VCME can occur in dogs with PLE. Unlike the five dogs with intestinal VCME, one dog with alimentary lymphoma did not demonstrate intestinal VCME, although hypoalbuminemia was suspected to be caused by PLE. In contrast, a case of VCME in the small intestine has recently been reported in a dog with *Toxocara canis* infection [18]. The dog in the previous report had no obvious hypoalbuminemia. It shows VCME may help identify enteropathy or PLE in early‐stage patients without significant hypoalbuminemia or relative clinical presentations. Unfortunately, dogs with enteropathy but without PLE could not be included in this study; therefore, it was difficult to determine whether intestinal VCME occurred in dogs with enteropathy but without PLE. Further studies are required to determine the specificity and sensitivity of intestinal VCME to canine enteropathy or PLE.

Alternative excretion of water‐soluble contrast agents has been reported as a common phenomenon in human medicine. VCME is more likely to be recorded with high doses of contrast agent for urography, which requires more passes through the body before the glomeruli filter it out [13], and with serious renal dysfunction or obstruction in patients with azotemia [4, 7, 19]. However, some authors have found VCME incidentally in patients with normal serum creatinine levels, and now it is considered a normal variant [20, 21]. Six dogs with VCME in the small intestine and cisterna chyli in this study had no elevated serum renal panel or urinary disorders. Furthermore, VCME was not observed in eight dogs that had azotemia or unilateral ureteral obstruction. VCME related to renal insufficiency could not be observed in this study.

Ultrasonographic hyperechoic mucosal striation is associated with lacteal dilation, having a sensitivity of 75% and a specificity of 96% for PLE [22, 23]. In the present study, ultrasonographic examinations were performed in three of five dogs with intestinal VCME. Hyperechoic mucosal striations were observed in two of these dogs, but not in one dog. Therefore, a delayed CT scan can be selected to provide additional diagnostic evidence for PLE. In contrast, hyperechoic mucosal striation can also be expected in dogs with no intestinal VCME; however, these dogs were not included in this study.

In the current study, VCME into the cisterna chyli was observed in one dog on delayed CT. Spontaneous contrast enhancement of the lymphatic system on delayed contrast‐enhanced CT was reported in feline patients undergoing intravenous administration of iodinated contrast medium [24], but not yet in dogs. The authors suggested that delayed CT can be added to obtain anatomical and functional information about the lymphatic system in cats [24]. In the present study, the spontaneous contrast enhancement of the lymphatic system in dogs was rarely observed, unlike in feline patients, and the cisterna chyli can be considered a rare alternative site of contrast medium excretion.

A delayed CT scan can be performed in the evaluation of the kidney, collecting system, and body trauma [25, 26]. It is not common to perform delayed CT scans, as it is not the gold standard for PLE diagnosis. However, the results of this study showed that intestinal VCME can be observed on delayed CT in patients with PLE. These results suggest that delayed CT can provide additional clues in certain situations, such as in cases where endoscopy and biopsy with histopathology cannot be performed.

The present study has several limitations. This study is not a systematic design and has a limited number of dogs with enteropathy or PLE, so the sensitivity and specificity of the CT finding of intestinal VCME for canine enteropathy or PLE could not be identified. Another limitation of the present study was that the final diagnosis by histopathology was not conducted in two of five dogs with intestinal VCME. Although VCME was observed even at 24 h after contrast administration in the previous reports of VCME in human medicine [2], additional delayed CT scans over 10 min were not done.

The presence of intestinal VCME on the delayed CT can be observed in dogs to be suspected of PLE, and it would be noninvasively used for additional supportive evidence of canine PLE before histopathologic evaluation, although the absence of intestinal VCME does not rule out PLE. Therefore, an additional delayed phase in multiphase CT examination should be obtained for the evaluation of dogs with intestinal diseases.

## List of Author Contributions

### Category 1

(a) Conception and design: S Choi
(b) Acquisition of data: Y Lee
(c) Analysis and interpretation of data: K Lee

### Category 2

(a) Drafting the article: Y Lee
(b) Revising article for intellectual content: H Choi, YW Lee

### Category 3

(a) Final approval of the completed article: S Choi

## Ethics Statement

Animal Usage Guidelines were followed for the study.

## Conflicts of Interest

The authors declare no conflicts of interest.

## Reporting Checklist

An EQUATOR network checklist was not used in this study.

## Previous Presentation or Publication

This study has not been published in another journal.
